# Yoga-based lifestyle intervention for antenatal depression (YOGA-D): study protocol for a pilot randomized controlled trial

**DOI:** 10.12688/wellcomeopenres.22493.2

**Published:** 2024-11-06

**Authors:** Rahul Shidhaye, Vidyadhar Bangal, Hemant Bhargav, Chitra Thanage, Suryabhan Gore, Shamal Talole, Kalyani Shinde, Swapnali Palande, Unnati Thete, Sonali Shelke, Geeta Gholap, Suchita Nisal, Soni Gargade, Swanand Tilekar, Nilam Behere, Kalpesh Game, Vaibhav Murhar, Rahul Kunkulol, Shirley Telles

**Affiliations:** 1 Department of Psychiatry, Pravara Institute of Medical Sciences, Loni, Maharashtra, India; 2 Department of Health, Ethics and Society, Care and Public Health Research Institute, Maastricht University, Maastricht, Limburg, The Netherlands; 3 Department of Obstetrics and Gynecology, Pravara Institute of Medical Sciences, Loni, Maharashtra, India; 4 Department of Integrative Medicine, National Institue of Mental Health and Neurosciences, Bengaluru, Karnataka, India; 5 Research and Development Cell, Pravara Institute of Medical Sciences, Loni, Maharashtra, India; 6 Community Representative, Loni, Maharashtra, India; 7 Center for Social Medicine, Pravara Institute of Medical Sciences, Loni, Maharashtra, India; 8 Public Health Foundation of India, New Delhi, India; 9 Independent Researcher, Bhopal, Madhya Pradesh, India; 10 Patanjali Research Foundation Trust, Haridwar, Uttarakhand, India

**Keywords:** Yoga, Preganancy, Depression, India

## Abstract

**Background:**

Depression during pregnancy is associated with pre-term labor, low birthweight, post-partum depression and adverse child outcomes. There are concerns about the safety of anti-depressant medications during pregnancy. Many pregnant women with antenatal depression are neither aware about their depression nor do they have access to non-pharmacological interventions for depression. Evidence suggests that pre-natal yoga can improve antenatal depression. Yoga is native to the Indian culture and women can practice yoga as a ‘self-care’ intervention with minimal training. There is no study till date on the efficacy of yoga on antenatal depression in pregnant women in a low resource (rural) setting in India. This pilot randomized controlled trial aims to study the feasibility, acceptability, and preliminary efficacy of a **YOGA**-based lifestyle intervention for Antenatal **D** epression (YOGA-D) in Maharashtra, India.

**Methods:**

We will undertake a single-blind individual randomized parallel group-controlled pilot trial with 1:1 allocation ratio. Adult women with 12–26 weeks of pregnancy, without any obstetric or medical complications will be randomly allocated to either the active intervention group (Yoga-Sanskar (YS)) or the Enhanced Usual Care (EUC) group. Trained yoga instructors will teach a pre-defined yoga sequence to the participants in the YS arm. In the EUC arm, participants will receive a single session of health education. We will assess trial feasibility using the recruitment, retention, and study completion rates. The primary outcome of depression will be measured using the translated Marathi version of the Patient Health Questionnaire-9. Assessments will be at the baseline, three-months post-randomization, and post-delivery.

**Discussion:**

This study will help us to understand the barriers in implementation of a yoga-based intervention for antenatal depression in a low-resource/rural setting in Maharashtra, India. Based on the learnings of this pilot trial, we plan to undertake an explanatory randomized controlled trial in the next few months.

**Registration:**

CTRI ( CTRI/2024/05/067176; 10/05/2024).

## Plain Language Summary

Pregnancy is not always a positive experience for all women. Some of them suffer from depression during pregnancy. This has negative impact on the health of an expectant mother, can lead to premature delivery, depression after pregnancy, and affects growth and weight of the fetus. Although women experience symptoms of depression, neither they are aware of this mental health condition, nor do they know what exactly needs to be done for the same. Anti-depressant drugs and ‘talk-therapies’ can be helpful, but the former may have adverse impact on fetal development and the latter is not widely available especially in rural settings in India. In this scenario, yoga practiced during pregnancy can be a potential solution. Women can practice yoga with minimal training, and it may have beneficial effects on depression during pregnancy. Through this study titled, ‘YOGA-based lifestyle intervention for Antenatal Depression (YOGA-D), our aim is to find out if it is possible for us to teach yoga to pregnant women in a rural setting in Maharashtra, India. Is yoga acceptable to them, will they practice yoga as per the plan we have finalized, and if they practice yoga regularly what effect will it have on their depression scores after three months of yoga practice and depression after delivery. YOGA-D is an experimental study where in half of the eligible women will be requested to practice yoga and the rest will continue with their routine care. Selection of the women in yoga group will be done randomly. Adult women (age>18 years) between 12 and 26 weeks of pregnancy, and who do not have any medical or pregnancy related problems are eligible to participate in the study. Depression will be measured using the Marathi version of the Patient Health Questionnaire-9. Stress, anxiety, and quality of life will also be measured.

## Introduction

### Background

Depression can affect women either during pregnancy (antenatal depression) or after the delivery (postnatal depression). Maternal depression is a serious public health problem and is associated with negative health outcomes both in women and their offsprings. In high-income countries, 7% to 15% women experience antenatal depression, in low-income and middle-income countries the prevalence of antenatal depression is higher (19–25%) ^1^. One in four women in South Asia is likely to experience depression during pregnancy, while in India, the pooled prevalence of antenatal depression is 17.7% (95% CI: 11.2%–26.9%) ^2^.

Antenatal depression is associated with higher risk of pregnancy-related mortality and morbidity, pre-term delivery, low birth-weight, post-partum depression and adverse neuro-cognitive developmental outcomes in infants, children and adolescents ^1^. Anti-depressant medications in pregnancy are associated with fetal exposure risk ^3^ and Cognitive Behavior Therapy based interventions are not yet scaled up due to several implementation barriers resulting in poor access to mental health care during pregnancy, especially in low resource settings ^4, 5^. To improve ‘access’ to care, there is a critical need to identify interventions that can be practiced by women as ‘self-care’, are culturally ‘acceptable’ to them, less resource-intensive and most importantly are ‘effective’ in treating antenatal depression.

Prenatal Yoga, a form of Yoga tailored to be safe, gentle, and helpful for pregnant women, represents a promising strategy for the prevention and treatment of antenatal depression in different settings across the globe. The findings of meta-analyses suggest that Yoga-based interventions are a promising non-pharmacological modality for the management of antenatal depression, and the overall safety, accessibility and low cost make it an appealing public health intervention ^6, 7^.

### Rationale for study

Yoga can be practiced by pregnant women as self-care with minimal training. Absence of evidence about efficacy (and cost-effectiveness) of a Yoga-based intervention to address antenatal depression in a low-resource rural and semi-urban Indian setting, presents a huge research gap.

### Objectives

The overall aim of this pilot randomized controlled trial is to examine the feasibility, acceptability, and preliminary efficacy of Yoga-Sanskar (YS), a yoga-based intervention to improve antenatal depression.

The primary objectives of this pilot randomized trial are:

1. To estimate participant eligibility, recruitment and retention-in-care and study completion rates.

2. To assess the feasibility of delivering Yoga-Sanskar intervention and acceptability of this intervention by the participants.

3. To assess the adherence rate, safety, and preliminary efficacy of Yoga-Sanskar intervention in improving antenatal depression (primary outcome).

The secondary objectives of this pilot randomized trial are:

1. To assess the preliminary efficacy of Yoga-Sanskar intervention on anxiety, stress, and quality of life (secondary outcomes) during pregnancy.

2. To assess the preliminary efficacy of the intervention on pregnancy outcomes (preterm labor, type of delivery, birthweight, and post-partum depression.

3. To explore the feasibility of administering all the evaluation tools including the Client Service Receipt Inventory to assess the healthcare and intervention costs.

4. To explore the feasibility of following up recruited participants four weeks post-delivery.

5. To study the psychometric properties of the translated versions of the Pregnancy Experience Scale, Common Symptoms in Pregnancy inventory, Attitude towards Yoga during Pregnancy scale, and the Five Facet Mindfulness Questionnaire.

***Trial design***

Single-blind individual randomized parallel group-controlled pilot trial with 1:1 allocation ratio.

## Methods

This trial protocol follows SPIRIT guidelines ^8^. Completed SPIRIT checklist is available as *Extended Data* ^9^.

### Research ethics approval

The Institutional Ethics Committee of the Pravara Institute of Medical Sciences (Approval Number: PIMS/DR/RMC/477) approved this study.

### Study setting

The study will be carried out in villages and towns close to Pravara Institute of Medical Sciences (PIMS) in Rahata and Shrirampur talukas of Ahmednagar district in Maharashtra, India. According to the 2011 census, Ahmednagar district has a population of 320,485 and is predominantly rural (81.8%). There are 14 administrative blocks (taluka) in Ahmednagar district, Rahata and Shrirampur are the taluka headquarters. PIMS is in Loni Budruk village about 20 kilometers south of Rahata and 25 kilometers west of Shrirampur town. PIMS has a tertiary care center with a 1275 bedded multi-disciplinary facility. The antenatal clinic of the Department of Obstetrics and Gynecology provides services to approximately 150–200 pregnant women every day. We will recruit pregnant women (residing in the study villages) from the antenatal clinic of the PIMS. Additionally, we will conduct small group meetings in the study villages to recruit pregnant women. The study villages are as follows: Loni Khurd, Loni Budruk, Kolhar Budruk, Kolhar-Bhagwatipur, Kolhar-Khurd, Babhaleshwar, Sakuri in Rahata taluka, and Gondhawani, Khandala, Belapur villages in Shrirampur taluka. We will also recruit participants from Rahata, Shirdi, and Shrirampur towns. We will approach obstetricians practicing in the above towns and if they agree then participants will be recruited from these private antenatal clinics.

### Eligibility criteria

We will first enlist all pregnant women in the study villages with the help of Anganwadi workers. We will invite these women to a small group meeting in the Anganwadi centers to explain the proposed study. Anganwadi is a community-based center providing healthcare check-up and referral health services, supplementary nutrition, immunization, and non-formal pre-school education. Expectant and nursing mothers, children, and adolescent girls are the primary beneficiaries. These centers were established as part of the Integrated Child Development Service (ICDS) and are managed by female community health workers called Anganwadi workers. Each center caters to a population of approximately 1000 people in rural areas. Anganwadi workers have an updated list of all pregnant women in their respective areas.

Similarly, trained Research Assistants (RAs) will approach pregnant women attending the antenatal clinic of the PIMS (and private antenatal clinics) and brief them about the proposed study. RAs will then screen and complete an eligibility assessment form for women who agree to participate in the study.

The inclusion/exclusion criteria are as follows:

***Inclusion criteria:***

- Adult pregnant women above 18 years of age
- Gestational age between 12–26 weeks
- Singleton pregnancy
- Patient Health Questionnaire-9 (PHQ-9) score of 10 or more
- Planning to stay in the study area (villages and towns described above) throughout study duration (approximately four months)

***Exclusion criteria:***

- Pregnant women advised rest/restriction of physical activities/laborious tasks by their obstetrician due to medical/obstetric problems
- History of one or more spontaneous abortions
- History of cervical stitch/cerclage operation
- History of pre-term labor/premature rupture of members
- Receiving treatment for depression or any other mental health condition
- Presence of death wish/ suicidal thoughts almost every day (score of 3 on PHQ9 item 9)
- History of hypertension, heart disease, diabetes (including gestational diabetes), respiratory disease, severe anemia, lower respiratory tract infection
- Inability to communicate in Marathi language
- Inability to attend in-person yoga sessions

Participants who completed the eligibility assessment but did not fulfill all the inclusion criteria will receive educational leaflets about physical activity, sleep, and diet during pregnancy.

### Informed consent

All eligible participants will receive a participant information sheet containing comprehensive details about the trial's objectives, study procedures, and contact information for the Principal Investigator and the Member Secretary of the Institutional Ethics Committee. An audiovisual rendition of this information sheet will be made available on the project website, and participants will be informed of the access procedure. We will provide ample time for the participants to review the information sheet and raise any queries regarding the trial before they are invited to participate.

Eligible participants who express willingness to participate in the study will provide informed written consent by completing a hard copy of the consent form ( *Extended Data* ^10^). Before signing the consent form, participants will have the opportunity to review the document and seek clarification about the study. For illiterate participants (unable to read and write Marathi but can communicate in Marathi), verbal consent (audio-recorded) and the thumb impression will be obtained after the RA reads the information sheet aloud. An impartial third party, such as a family member or healthcare worker, will witness the consent process and sign the consent form.

We will seek separate written informed consent at the three-month follow-up assessment, post-delivery assessment, and for participation in the qualitative study and for audio/video recording of yoga sessions to evaluate intervention fidelity and quality.

RAs will undergo training to ensure a comprehensive explanation of the study's purpose, randomization, and other procedures to the participants and to ensure the proper acquisition of informed consent. They will also be required to sign the consent forms.

Participants will receive a copy of the participant information sheet. Those who decline participation will be asked for the reason for refusal, and with their consent, their age, education, occupation, and religion will be documented. We will also record the refusals during the three-month and post-delivery follow-up assessments.

### Interventions

In this section, we will first describe the active intervention (Yoga-Sanskar (YS)) followed by the description of the Enhanced Usual Care (EUC) that will constitute the comparison arm. This description will answer the following questions: 1) What is the rationale for the YS intervention? 2) What are the key components of the YS intervention? 3) How will yoga instructors teach the intervention, and how will it be practiced by the participants?

We have followed Template for Intervention Description and Replication (TIDieR) guidelines to describe both the interventions ^11^.

***Intervention arm: Yoga-Sanskar intervention for antenatal depression***

YS is a type of gentle, prenatal yoga-based intervention. It is a practice of micro-circulation/body-loosening exercises ( *sukshma-vyayam*), postures ( *asanas*), breathing exercises ( *pranayama*), and relaxation ( *shavasana*) by pregnant women to improve their mental and physical health. YS intervention has two components: a core yoga sequence and several behavioral strategies to improve home practice (or self-practice) of the core yoga sequence.

***Rationale for the intervention***

The core yoga sequence in the Yoga-Sanskar intervention is an adapted version of the Integrated Approach for Yoga Therapy during Pregnancy (IAYT-P) ^12^. Researchers from the Swami Vivekanand Yoga Anusandhan Kendra have developed and evaluated this intervention in an urban population in the southern part of India. Butzer *et al.* have proposed a model to explain the beneficial effects of yoga practice (postures, breathing, relaxation, meditation) on mood, well-being, quality of life, and positive behaviors ^13^. According to the authors, the practice of yoga improves mind-body awareness (increased mindfulness and self-awareness), self-regulation (better emotional and self-regulation), and physical fitness (increase in physical self-efficacy, strength, balance), leading to positive outcomes ^13^. In pregnant women, regular yoga practice may likely reduce stress by the above mechanism and through modulation of the hypothalamic-pituitary-adrenal axis activity. While adapting the intervention, we realized that yoga is a form of behavior change. Hence, our intervention first tries to improve the capability of the participants to practice yoga, provides them an opportunity to engage in regular practice, and tries to build their motivation to continue practicing yoga for a healthy behavior change (COM-B) ^14^. We will assess the minor ailments experienced by pregnant women at the baseline (details below) and propose to them to practice yoga to address these problems. This approach will directly address the 'felt need' of the participants and improve their motivation to practice yoga. Participants are likely to experience several barriers while practicing the intervention. We tried to address these barriers by developing a Theory of Change map with the community health workers and pregnant women that includes behavioral strategies to improve adherence to home practice of yoga.

The two phases of the intervention, LEARN and PRACTICE, are inspired by the approach used in the World Health Organization's mYOGA mobile application ( https://www.who.int/initiatives/behealthy/who-myoga-app)

***Components of the intervention***

The two phases of the intervention are the LEARN and PRACTICE phases.

In the LEARN phase, participants randomized to the intervention arm will be taught the entire core yoga sequence in a directly supervised group yoga session format by a trained yoga instructor. The first group session (Commitment building/ *Yoga-Nischaya Satra*) will be on the day of randomization, where the yoga instructor will give a brief overview of the entire program spread over three months, discuss potential benefits of yoga practice, safety concerns, and most importantly will emphasize on the commitment to learning and practicing yoga. In this session, the yoga instructor will demonstrate the entire yoga sequence, and participants will also get a chance to practice relaxation and a few other yoga activities. Three directly supervised group yoga sessions will follow the first session, preferably on three consecutive days, where the participants will learn all the activities included in the yoga sequence. The group yoga session will be for 60 to 75 minutes. In a typical session, participants will first complete the safety checklist that is designed based on the guidelines of the American College of Obstetricians and Gynecologists ^15^. Participants experiencing any warning signs in the checklist will be requested not to practice yoga. The yoga instructor will sign off the safety checklist. The session will begin with an introduction of the participants and a brief overview of the session by the yoga instructor. Participants will be taught *sukshma-vyayam* (micro-circulation exercises), *asanas* in standing, sitting, supine position, *pranayama*, and *shavasana*. The yoga sequence to be practiced in the second and third trimesters will differ. Table 1 provides a detailed description of the yoga session and the activities included in the second and third trimesters. During the sessions, yoga instructors will provide particular emphasis on

1) awareness of breath

2) focus on bodily sensations during each of the yoga activities

3) necessary precautions to avoid injuries due to yoga practice.

**Table 1.** Yoga-Sanskar Intervention.

	Type of Activity	Duration (Second Trimester)	Duration (Third Trimester)
**1.**	**Opening Prayer**	**1 minute**	**1 minute**
**2.**	***Sukshma-Vyayam*** **(Micro-circulation ** **exercises)**	**10 minutes**	**10 minutes**
	*Griva Sanchalan* Neck movements	Yes	Yes
	*Hasta Ayama Svasanam* (Hands in and out breathing)	Yes	Yes
	*Hasta Vistara Svasanam* (Hands stretch breathing)	Yes	Yes
	*Vyaghra Svasanam* (Tiger breathing)	Yes	No
	*Setu Bandha Svasanam* (Bridge posture breathing)	Yes	No
**3.**	***Asana* (Postures)**	**15 minutes**	**15 minutes**
	**Standing *Asanas***		
	*Tadasana* (Tree pose)	Yes	Yes
	*Ardhakati Chakrasan* (Lateral arc pose)	Yes	Yes
	**Sitting *Asanas***		
	*Vajrasana* (Ankle pose)	Yes	Yes
	*Siddhasana* (Sage pose)	No	Yes
	*Baddhakonasana* (Bound ankle pose)	No	Yes
	*Upavistakonasana* (Spread legs pose)	No	Yes
	*Malasana* (Garland pose)	No	Yes
	**Supine *Asanas***		
	*Viparita Karani* (Half shoulder stand)	Yes	No
	*Supta-baddha Konasana* (Folded leg lumbar stretch)	Yes	Yes
**4.**	***Pranayama and* Meditation**	**10 minutes**	**10 minutes**
	*Vibhagiya Pranayam* Sectional breathing	Yes	Yes
	*Anulom-Vilom Pranayam* (Alternate nostril breathing)	Yes	Yes
	*Bharamari* (Humming breath)	Yes	Yes
	*Nadanusandhana* (Mind-sound resonance)	Yes	Yes
**5.**	***Shavasana*** **Relaxation**	**10 minutes**	**10 minutes**
**6.**	**Closing Prayer**	** 1 minute**	** 1 minute**
	**Total Duration**	**47 minutes**	**47 minutes**

After the session, the yoga instructor will spend time with each participant individually and suggest changes/modifications based on her observation and any problems/difficulties the participants might have in practicing yoga-based activities. After the end of these four sessions, participants are expected to practice yoga independently for three months. Participants will appear for assessment within three to four weeks after the first session to ensure they have gained adequate competency to practice yoga. The details about the competency assessment are provided below. Weekly group yoga sessions will follow to address the participants' concerns and problems during the yoga practice. These sessions will also help to keep participants motivated to practice yoga.

Currently, we have two yoga instructors as part of our team, and both are co-authors of this paper (GG and SN). GG received her training as yoga instructor from Yoga Vidyadham, Nashik, is practicing and teaching yoga to general population for eight years and to pregnant women for the last five years. SN has completed basic training as a yoga instructor from Arogyavardhini Yoga Nisargopachar Kendra, Ahmednagar and is teaching yoga to general population and pregnant women for the last two years. Both have appointments with the government public health system under the initiative by the AYUSH ministry and regularly conduct yoga sessions for pregnant women and members of the general population in the newly established Ayushman Arogya Mandirs (Health and Wellness Centers). We will conduct an orientation session for the yoga instructors before the start of the trial. Yoga instructors are aware of the yoga sequence included in the current study. In the orientation session, we will explain to them the structure of a typical yoga session with particular emphasis on participant safety. Warning signs included in the safety checklist will be explained to them in detail and they will advise participants not to practice yoga if any of the warning signs are present. Yoga instructors will be trained on strategies to improve adherence to home practice of yoga.

The PRACTICE phase will begin after the first group session alongside the LEARN phase. We will encourage participants to start practicing as soon as possible. In the initial three weeks after the recruitment, we will request participants to practice at least one activity from the yoga sequence. After completing the competency assessment, around the second month onwards, participants will be encouraged to practice the entire yoga sequence daily (or at least half the sequence). Multiple tools will be provided to the participants to facilitate the home practice. Yoga Journal ( *Yoga Dainandini*) will be the most important of these tools. It will contain a detailed day-to-day plan of home practice and images of yoga activities to be practiced each day. A QR code will be provided along with the image, which will link to the YouTube video of that yoga activity. The journal will also have a section to track the participants' mood, anxiety, irritability, sleep, and fatigue. A yoga manual with a detailed description of each yoga activity in the yoga sequence will be provided to the participants. This will help them see the activity on YouTube, read the description from the manual, and then practice it independently. Additional reading material will be provided to them that will contain the details related to safety and the potential benefits of yoga. In addition to the yoga practice, participants will receive educational leaflets about physical activity, sleep, and diet during pregnancy.

A local community member (female) whom we have named *'Sahayogini'* will regularly meet the participants to motivate them to practice yoga at home using the schedule given in the journal. They will discuss barriers to yoga practice and try to find solutions through one-on-one discussions with participants. *Sahayoginis* will also help organize and facilitate weekly yoga sessions.

***Comparison arm: Enhanced Usual Care (EUC)***

Participants randomized to the EUC arm will receive a single health education session delivered by the Intervention Coordinator (IC). During this session, aspects related to sleep hygiene and diet during pregnancy will be discussed, and they will be encouraged to undertake regular physical activity (150 min/week). They will be provided educational leaflets containing information about physical activity, sleep, and diet during pregnancy. The IC will work with the participants to identify ways to reduce stress during pregnancy and improve social support. As per the current standard of care in the antenatal clinic of Department of Obstetrics, pregnant women are neither screened for stress nor is there any discussion about improving mental health during pregnancy. We will also offer a slightly modified yoga-based intervention in the post-partum period (six weeks after delivery) to participants in the comparison arm.

***Criteria for discontinuing or modifying allocated interventions***

If there is spontaneous abortion or stillbirth, participation in both arms will be discontinued. Other than this, there are no specific criteria to discontinue the intervention. A few modifications in the sequence for individual participants may be made by the yoga instructor based on feedback from them. Obstetricians may advise participants to discontinue yoga due to health issues, or they may decide to stop practicing yoga themselves for any reason.

***Strategies to improve adherence to interventions***

The IC, yoga instructors, and *Sahayoginis* will regularly contact participants in the YS arm to motivate them to practice yoga at home regularly. Adherence to home practice will be assessed using the yoga journal. We expect *Sahayoginis* to contact participants at their homes at least once a week. They will also contact participants in the EUC arm at least once a week by phone. This will help reduce attentional bias.

***Relevant concomitant care permitted or prohibited during the trial***

Participants will receive usual care throughout the trial, and no specific component of antenatal care or other health services will be prohibited.

***Provisions for post-trial care***

Participants in both arms will receive information about yoga sessions they can join after the end of the trial. Participation in yoga sessions post-trial will be entirely voluntary. No compensation will be provided to participants who suffer non-negligent harm from trial participation.

***Outcomes***

We will assess the feasibility and acceptability of the Yoga-Sanskar (YS) intervention and the preliminary efficacy of the intervention on depression in pregnant women.

Process data will inform the feasibility of the enrolment, engagement with the intervention, and completion of the intervention. The process indicators are as follows:

1. Number and proportion of eligible participants enrolled per week in the YS and the EUC arm.
2. Number and proportion of enrolled participants in the YS arm who attend the first session, three follow-up training sessions, and the final assessment session.
3. Number and proportion of enrolled participants in both arms who complete the follow-up assessment three months post-randomization.

Additionally, we will assess the feasibility of following up with participants until delivery and conducting all the assessments four weeks after delivery.

RAs in the trial evaluation team will maintain the study enrollment logs, and the IC and *Sahayoginis* will maintain the intervention delivery log. The logs will be in paper format, and at the end of every week, RAs and IC will enter all the data on an access-restricted computer. We will save the data in password-protected CSV files. Process data for feasibility assessment will be obtained from the above logs.

Trial participants will complete a structured satisfaction survey at the end of the training and the final assessment sessions. We will conduct In-Depth Interviews with trial participants and yoga instructors. These interviews will focus on the intervention’s delivery, content, comfort, and complexity. We plan to enroll around 20 participants (12-13 from the YS arm and 7-8 from the EUC arm). However, data saturation will determine the final sample size for the qualitative study. Quantitative data obtained from the satisfaction survey (details below) and qualitative data from the IDIs will inform the acceptability of the intervention.

The description of measures that will be used to assess the preliminary efficacy of the intervention is given in Table 2.

**Table 2.** Measures to assess the preliminary efficacy.

Outcome	Measure	Time-point
Depression	Patient Health Questionnaire-9	Baseline, One, two, and three-months post-randomization, and post-delivery
Anxiety	Generalized Anxiety Disorder-7	Baseline, three-months post-randomization, and post-delivery
Stress	Perceived Stress Scale (PSS)	Baseline, three-months post-randomization, and post-delivery
Quality of Life	EuroQoL 5 Dimensions Score (EQ-5D-5L)	Baseline, three-months post-randomization, and post-delivery
Minor ailments in pregnancy	Common Symptoms in Pregnancy (CSIP)	Baseline, three-months post-randomization, and post-delivery
Experience of pregnancy	Pregnancy Experience Scale	Baseline, three-months post-randomization, and post-delivery
Attitude	Attitude towards Yoga practice during pregnancy scale	Baseline, three-months post-randomization, and post-delivery
Treatment Expectation	Credibility Expectancy Questionnaire	Three to four weeks post-randomization
Self-Efficacy	Yoga Sequence Self-Efficacy Assessment Yoga Practice Self-Efficacy Assessment	Three to four weeks post-randomization
Competence	Yoga Performance Assessment Scale Online Structured Competency Assessment	Three to four weeks post-randomization
Awareness	Five Facet Mindfulness Questionnaire	Baseline, three-months post-randomization, and post-delivery
Satisfaction	Structured Questionnaire	Three to four weeks post-randomization and 3-months post- randomization
Home Practice	Self-report log	Every day
Injuries due to yoga	Self-report log	Every week for Three-months post-randomization
Serious Adverse Events (SAE)	SAE report form	Baseline, three-months post-randomization, and post-delivery

***Depression***

Depression during pregnancy is the primary outcome to assess the efficacy of the intervention. We will assess depression using a culturally adapted and validated Marathi version of PHQ-9. PHQ-9 is a nine-item tool to screen depression based on the Diagnostic and Statistical Manual-IV criteria (DSM-IV) ^16^. The Marathi version of the tool is in the supplementary file ( *Extended Data* ^17^).

***Anxiety***

GAD-7 is a seven-item tool to screen anxiety, also based on the Diagnostic and Statistical Manual-IV criteria (DSM-IV) ^18^. The Marathi version of the tool is in the supplementary file ( *Extended Data* ^17^).

***Stress and Social Support***

We will use a shorter (10-item) version of the original Perceived Stress Scale with 14 items ^15^ to assess perceived stress and the Multidimensional Scale of Perceived Social Support to assess perceived social support ^19^. PSS-10 measures participants' appraisal of various life situations in the last month and how unpredictable, uncontrollable, and overloaded they felt due to these situations. PSS and MSPSS are widely used in research studies across the globe, and they have acceptable psychometric properties ^20, 21^. In this study, we will use the Marathi version of the PSS and the MSPSS that were translated from the English versions using the World Health Organization (WHO) guidelines to translate and adapt instruments ^22^. The Marathi versions of the tools are uploaded in the supplementary file ( *Extended Data* ^17^).

***Quality of Life***

We will measure Health-Related Quality of Life (HRQoL) using the European Quality of Life Five Dimension (EQ-5D-5L) ( https://euroqol.org/eq-5d-instruments/all-eq-5d-versions/) descriptive system. We will generate a single utility score using the value set/preference weights for the Indian population ^23^. Participants will also rate their overall health using a visual analog scale (VAS). We will use the Marathi version of EQ-5D-5L with permission from the EuroQoL Group ^24^. The EQ-5D has been validated in different parts of the world ^25^.

***Minor ailments during pregnancy***

Pregnant women reported multiple physical, psychological, and interpersonal problems during the in-depth interviews and in the intervention development workshops carried out during the formative research phase. Non-life-threatening minor illnesses/ailments during pregnancy have a very high prevalence and they impact lives of pregnant women in a big way ^26^. We developed a 51-item Common Symptoms in Pregnancy (C-SIP) inventory to assess these minor ailments. Women will be requested to select the symptoms they are experiencing, rate them on a scale of 1–10 (1-very less, 10-very high), and comment on whether they think that the symptoms/ailments need any treatment. The Marathi version of C-SIP is in the supplementary file ( *Extended Data* ^17^).

We will use these symptoms to assess the ‘Minimal Clinically Significant Difference’. Participants in both arms will identify the symptoms at the baseline and the beginning of each week. They will report improvement/worsening in these symptoms at the end of the week. Women will attribute the change in the symptoms to yoga practice (YS arm) or to any other intervention they undertake (EUC arm) to address these symptoms.

***Pregnancy experience***

Being pregnant was a positive experience for many women who participated in the in-depth interviews as part of the formative research. The role of positive emotions cannot be overlooked as we focus on stress, anxiety, and depression during pregnancy. We will use a 20-item brief version of the Pregnancy Experience Scale-Brief Version to assess ten uplifts and ten hassles experienced by women during pregnancy. Each item of this scale will be rated on a 4-point Likert scale for both uplifts (how much the item makes the participant feel “happy, positive, or uplifted”) and hassles (how much the item makes the participant feel “unhappy, negative, or upset”) ^27^. The original 41-item scale is modeled on the Hassles and Uplifts Scale ^28^. We will use the culturally adapted and translated Marathi version of the PES-Brief. The Marathi version of PES-Brief is in the supplementary file ( *Extended Data* ^17^).

***Attitude towards Yoga***

The intervention will affect outcomes only if the intervention is regularly practiced by the participants/pregnant women. The attitude of pregnant women toward yoga practice will determine if they can adopt a new behavior and practice yoga regularly. Affective, behavioral, and cognitive are the three components of attitude. We will assess these using a newly developed 'Attitude towards Yoga Practice during Pregnancy' (AYP) scale. The scale consists of items that try to understand pregnant women's awareness of prenatal yoga, the perceived benefits, and harms of practicing yoga during pregnancy, their feelings towards yoga, and the anticipated barriers that can affect the regular yoga practice. The Marathi version of AYP is in the supplementary file ( *Extended Data* ^17^).

***Self-efficacy***

Self-efficacy is an individual’s belief in his/her ability to perform a specific behavior ^29^. Participants will regularly practice yoga if they consider that they can perform all the activities suggested in the yoga sequence and overcome the barriers to daily yoga practice at home. We developed Yoga Sequence Self-Efficacy Assessment Tool (YS-SEAT) and Yoga Practice Self-Efficacy Assessment Tool (YP-SEAT) to assess self-efficacy. In YS-SEAT, there are a total of 19 activities included in the yoga sequence. At the end of the LEARN phase (three to four weeks post-randomization), participants will rate their ability to perform each of these 19 activities on a five-point scale (one will not be able to do at all, five will be able to do very well). In YP-SEAT, participants will rate their confidence in practicing yoga regularly at home despite the existing barriers. Our formative research informed the inclusion of the six barriers that women generally face in the study context: fatigue/tiredness, guests at home, childcare, boredom, household work, lack of space at home. For each of these six barriers, participants will rate their confidence on a five-point scale (one will be no confidence at all, five will be full (100%) confidence). These tools are adapted from the Yoga Posture Self-Efficacy Assessment and Yoga Practice Adherence Self-Efficacy respectively, developed by Nicosia *et al.* ^30^. The tools are in the supplementary file ( *Extended Data* ^17^).

***Competence***

The yoga instructor needs to assess the ability of the participants to practice the entire yoga sequence in addition to the self-reported ability by the participants. Participants will perform the entire yoga sequence independently in the fifth supervised yoga session, which will be the last session of the LEARN phase. The yoga instructor will rate the performance of each of the participants using Yoga Performance Assessment scale developed by Hariprasad *et al.* ^31^. Individual yoga activities will be first rated on a scale of 0–3 (0-Can’t practice at all, 1-Needs assistance throughout the practice, 2-Needs assistance through some steps of the practice, 3-Can practice with ease, without assistance of instructor). This will be followed by an overall rating on parameters like (a) ability to complete the entire yoga sequence, (b) ability to remember and complete each step of the yoga practice and remember the sequence, (c) ability to co-ordinate breathing with *yoga-asana*, (d) ability to practice *pranayama* as per the instructions and the ability to chant AUM correctly (e) ability to relax during *shavasana*.

Participants will also complete an online structured competency assessment before the fifth supervised yoga session. As part of this assessment, they must identify the names of all the yoga activities, the appropriate sequence of the included activities, the benefits of key *asanas* and *pranayama*, and how to ensure safety during yoga practice. The assessment will have 40 points and the participants must get a full score on this assessment. They can take the assessment multiple times till they get the full score.

***Satisfaction***

We have developed a structured feedback form for the participants to express their satisfaction with the supervised group yoga sessions and other components of the Yoga-Sanskar intervention (Journal, dashboard, YouTube videos, yoga sequence chart, competency assessment, and interaction with *Sahayoginis*). Participants will provide ratings on their overall experience of the supervised group yoga sessions, the information provided prior to the sessions, the consenting process, various activities in a group yoga session, the opportunity to ask questions and interact with yoga instructors, the style of teaching, duration of the sessions, and the overall organization of the sessions. The rating will be on a five-point scale (1-very bad, 2-bad, 3-can’t say, 4-good, 5-very good) and will be completed at the end of the LEARN phase.

***Treatment expectation***

A participant’s belief about the efficacy of a therapy/behavioral intervention has a cognitive and an affective component. Credibility is the cognitive component and is defined as ‘how believable, convincing, and logical the treatment is’, while the expectancy is the affective component, and it captures the participants belief about the improvement that will be achieved by the intervention ^32^. Credibility and expectancy play an important role in determining the outcomes of a therapy/behavioral intervention. We developed a Marathi version of the Credibility Expectancy Questionnaire for this study ^33^. Participants will complete this at the end of the LEARN phase. The Marathi version is in the supplementary file ( *Extended Data* ^17^).

***Fidelity of the intervention delivery***

We will assess the fidelity of the intervention delivery based on the checklist completed by the yoga instructors. For each session, the instructor will give a rating on a scale of 0–3 for the following items: self-introduction and introduction of participants with each other, checking with participants about difficulties in practicing yoga (day 2 onwards), outline of the session provided, health benefits of yoga, aspects related to yoga philosophy, awareness, and safety discussed, all components of yoga sequence explained and demonstrated, advise on sleep, hygiene, and diet provided, discussion with individual participants done, and plan for home practice explained. We will primarily depend on the instructors’ self-report as it is very challenging to video record the sessions in a community setting. We will assess the feasibility of video recording yoga sessions and undertaking independent observation by a study team member.

***Self-awareness/mindfulness***

Improvement in self-awareness/mindfulness is proposed as a potential mechanism of action through which a yoga-based intervention improves mental health outcomes/depression ^13^. The training during yoga sessions will focus on improving self-awareness/mindfulness, as the increase in mindfulness is associated with decreased distress and other psychological symptoms. We will use a 17-item Five-Facet Mindfulness Questionnaire that measures five aspects: non-reactivity to inner experience, observing, acting with awareness, describing with words, and non-judging of experience. The original English version of the scale ^34^ is translated into Marathi and is included in the supplementary file ( *Extended Data* ^17^).

***Pregnancy outcomes***

We will gather information about the outcome of pregnancy (normal birth/stillbirth), whether the delivery took place at-term, and the birthweight of newborn based on the hospital records.

***Self-report log***

Participants in both arms will document any injuries or non-serious adverse events during the trial in the yoga journal (and a similar journal will be given to the participants in the EUC arm). IC and *Sahayoginis* will keep track of this and report to the CRC in case of adverse events.

***Economic evaluation***

In the future explanatory trial, we plan to assess the cost-effectiveness of the YS intervention relative to the EUC. Effectiveness/health outcomes will be estimated using the Quality Adjusted Life Years based on the EQ-5D-5L data collected at the baseline and three months post-randomization. Costs will comprise intervention costs, healthcare resource costs, and wage loss costs. In this pilot trial, we will explore the feasibility of using the Client Service Receipt Inventory to estimate healthcare resource costs and wage loss costs. Intervention costs will be estimated using the trial records.

### Participant timeline

**Figure 1.**
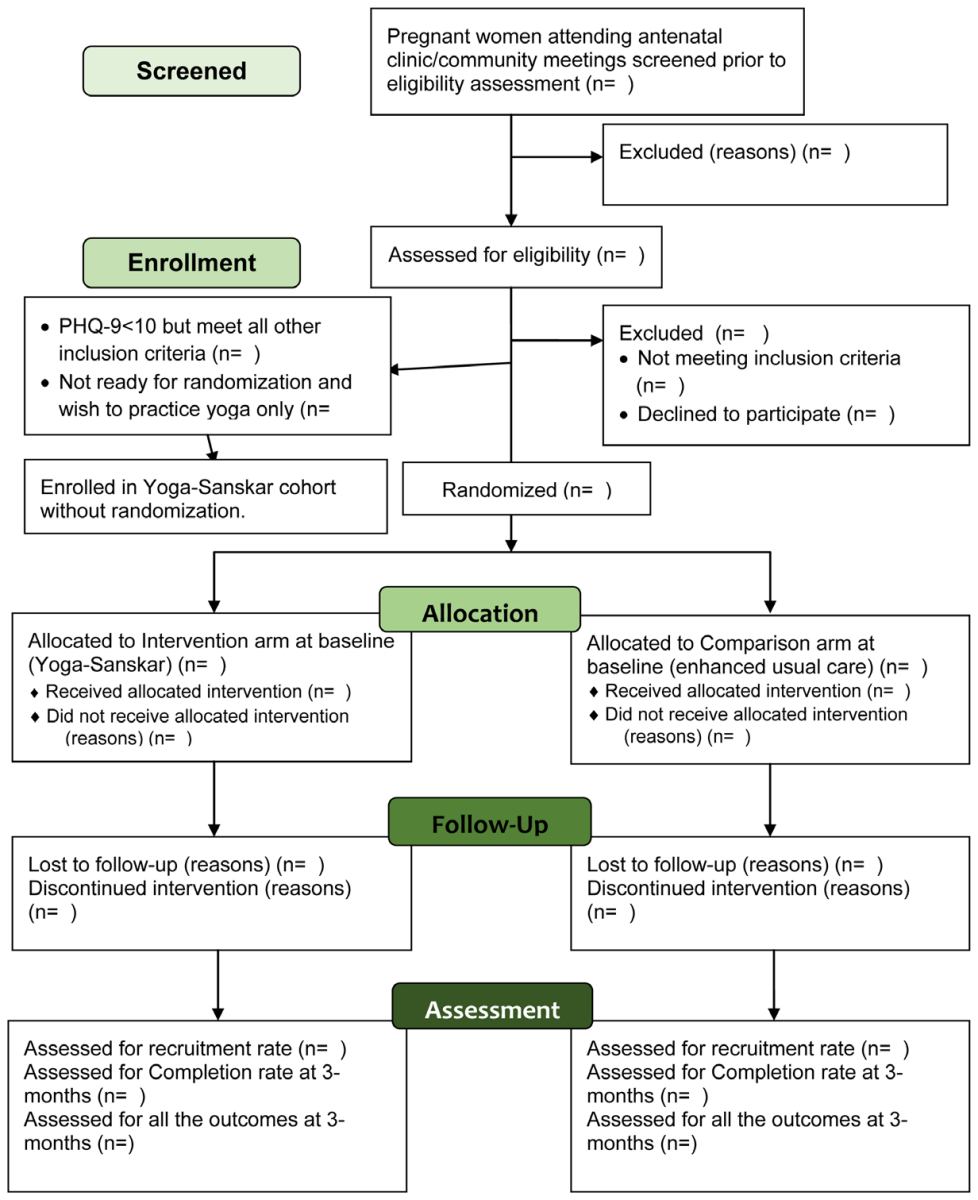
Participant flow diagram through YOGA-D pilot randomized trial.

Participant’s timeline through the trial is presented in Figure 1.

### Sample size

We plan to recruit around 25 pregnant women with depression in each arm (total=50). This is based on the recommended sample size for pilot randomized controlled trials by Whitehead *et al.* According to the authors, a sample size of 25 in each arm is adequate for a small standardized effect size (0.2) between the two arms ^35^. Assuming a conservative prevalence of 10% for antenatal depression and a 15% non-response rate, we plan to screen around 600 pregnant women.

### Recruitment

Participants will first complete the informed consent procedure; the baseline assessment will follow this. RAs will administer the structured questionnaire before the randomization. The structured questionnaire will include data on demographic and socio-economic measures and primary and secondary outcome measures. Informed consent procedure and baseline assessment will take place in the community setting (Anganwadi or a public health facility) or antenatal clinic of PIMS. Participants eligible for trial enrollment will be requested to come to the community/health center closest to their homes in about a week for randomization. IC will randomize the participants in the community/health center, and he/she will work independently of RAs who will not be aware about the treatment allocation.

It is likely that some women may wish to practice yoga and may not be ready for randomization, while some may also wish to practice yoga, but their PHQ-9 score is less than 10. We will offer them an alternative to be part of a cohort and provide the Yoga-Sanskar intervention. We will follow them similarly to the trial participants and complete the three-month and post-delivery outcome assessments.

### Assignment of interventions: allocation

***Sequence generation***

Serially numbered, opaque, sealed envelopes (SNOSE) will be used for treatment allocation. Clinical Research Coordinator (CRC) based in the Directorate of Research, PIMS will prepare SNOSE using the randomization list created by the trial statistician who will independently generate the randomization list using the statistical program R (version 3.6.1). First, a list of variable block sizes (randomly selected block sizes of 2/4/6/8) will be created, followed by a list of intervention allocation (YS or EUC) using a 1:1 allocation ratio. CRC and trial statistician will be independent of the study team and will not interact with the RAs or the IC.

***Allocation concealment mechanism***

The IC will randomly allocate participants to the intervention group using SNOSE after they complete their baseline assessment.

***Implementation***

The IC will collect a set of opaque sealed envelopes from CRC as per the serial numbers a few hours before the recruitment. The IC will open the opaque sealed envelopes (as per the serial numbers) in front of the participants and immediately inform them about the intervention group (i.e., YS or EUC) assigned to them. Participants will be asked to keep their group assignment private from the RAs at the three-month follow-up, and post-delivery assessments. To maintain allocation concealment, neither the RAs nor any staff working at the hospital will have access to the randomization lists. The IC will inform the CRC of the unique identification number of the participant (and no other details) and the intervention allocation group. The CRC will maintain the record of intervention allocation.

### Assignment of interventions: blinding

***Who will be blinded?***

Participants, their family members, and study team members involved with intervention delivery (IC and yoga instructors) will be aware of participants’ assigned intervention during the trial. It is not possible to avoid bias arising due to this. RAs will complete baseline, three-month follow-up, and post-delivery assessments, and they will not interact in any way with the intervention team. RAs will not be aware of the intervention allocation status. Participants and family members will be instructed not to disclose whether they are receiving YS intervention or EUC to the RAs at the time of the follow-up assessments. Additionally, RAs will also remind participants not to reveal their group assignment to them so that they remain blind to the participant’s treatment allocation.

Obstetricians will not be aware of the intervention allocation status, minimizing the risk of any change in their behavior while dealing with the participants.

The intervention team led by the IC and the outcome assessment team comprising RAs will not have any interactions during the trial. They will work independently of each other in separate locations and will have no communication about trial participants and their intervention allocation status. During the training of RAs in the outcome assessment team, particular emphasis will be given to explaining the equipoise between the two interventions.

We will monitor the risk of contamination at the level of the yoga instructors, intervention coordinator, and participants. Yoga instructors and the intervention coordinator will be requested not to share the information about YS with pregnant women other than those enrolled in the intervention arm. They will also ensure that participants in both the arms adhere to the group randomly allocated to them.

***Procedure for unblinding if needed***

If there is any yoga-related adverse event, we will reveal the intervention group allocation to the obstetrician.

### Data collection and management

***Plans for assessment and collection of outcomes***

Research Assistants (RAs) will utilize Case Record Forms (CRF) to gather information during the initial assessment and the three-month follow-up. Data collection will be conducted using pen and paper, with subsequent manual entry into Microsoft Excel for digital conversion. The data will be stored as a CSV file, with original timestamps for entries and a record of any subsequent modifications in an audit trail. Documentation concerning the fidelity and quality of yoga intervention delivery will follow a similar process, with data stored in CSV format. Qualitative data will be recorded using digital voice recorders, and accompanying memos and field notes will be transcribed from Marathi to English for analysis. Data will be anonymized but associated with the trial ID.

The Clinical Research Coordinator (CRC) will conduct weekly checks on each data source for accuracy, consistency, and completeness, promptly addressing any queries and updating the database while maintaining an audit trail. Initially, separate databases will house distinct data types, with consolidation into a master database upon completion of data collection. Password protection will safeguard the data, with exclusive access granted to the CRC and no other trial team members.

***Plans to promote participant retention and complete follow-up***

During the initial assessment, RAs will inform the participants about the date for the three-month follow-up evaluation. RAs will call the participants one week before the follow-up assessment to remind them about the assessment. If the participant is traveling or is unavailable for any reason, there will be a seven-day window following the due date to complete the assessment. The assessments will be conducted preferably at the health center or Anganwadi center. If the participant is unable to come there, then the RA will complete the assessment at participant’s home.

***Data management***

We will prepare a Trial Master File that will encompass all vital documents. Documentation pertinent to the trial will be preserved for seven years following its conclusion. After this period, paper-based data will be securely disposed of, while electronic data will be purged of identifiable information.

All Case Record Forms (CRFs) and associated study documents will be securely housed within the Directorate of Research, PIMS. Password-protected servers will safeguard electronic records. We will use coded identification numbers to identify data, ensuring participant confidentiality. Participant information in physical form will be stored securely in locked cabinets. We will utilize anonymized data to analyze and disseminate results at conferences and through academic publications.

***Confidentiality***

The identity and privacy of research participants will be protected. Any variable that may help infer the identity of any participant will be deleted from the database.

### Statistical methods

We will first compute the descriptive statistics for demographic, socio-economic, pregnancy-related characteristics, and outcome measures for all the trial participants in both arms. Descriptive statistics will include means and standard deviations (for continuous variables) or proportions (for discrete variables). We will use a t-test and chi-square test to compare means and proportions, respectively, to estimate the difference in the baseline characteristics between the two arms. We will use the intention-to-treat analysis to assess preliminary efficacy, binomial regression models to estimate risk ratios and 95% confidence intervals for binary outcomes and linear regression models to estimate regression coefficients for continuous outcomes. Analyses of continuous outcomes will additionally adjust for the baseline value of that outcome and any imbalances of baseline values of key covariates. The trial statistician will conduct the statistical analyses in Stata version 18.

***Methods for additional analyses (e.g., subgroup analyses)***

*Process evaluation analysis*

We will audio-record all the in-depth interviews (IDIs) and focus group discussions and maintain field notes. Interviews in English will be transcribed verbatim, while those conducted in Marathi will be transcribed and translated into English. Team members proficient in both English and Marathi will conduct back-translation checks. Qualitative data will undergo analysis using the content analysis method. An initial coding framework comprising overarching themes will be established a priori. Subsequent themes will emerge from the data through inductive reasoning. NVivo 9 software (QSR) will facilitate data coding. The Principal Investigator will primarily oversee data analysis and synthesis of findings.

***Methods in analysis to handle protocol non-adherence and any statistical methods to handle missing data***

Complier Average Causal Effect (CACE) analysis will be carried out to assess the effect of receiving treatment as defined in the protocol. CACE will be done in two ways. Full compliance is defined as attendance at the first five sessions and completing all the competency assessments. The first type of analysis will involve fully compliant participants. In the second type of analysis, participants with any compliance (attendance at one yoga session or more) will be included. The extent and pattern of missing data for each outcome will be explored. Multiple imputation of missing values will be performed if there are more than 10% missing values at endpoint.

***Plans to give access to the full protocol, participant level data and statistical code***

The trial protocol will be submitted to an open access journal before the initiation of participant enrollment. Additionally, the trial protocol and the supplementary files will also be uploaded on a dedicated website:

https://rahulshidhayelab.in/YOGA_D/

Six months after the completion of the trial, data will be uploaded on an open access data repository (e.g., Figshare) along with the statistical code used for the analysis.

### Oversight and monitoring

***Composition of the coordinating center and trial steering committee***

The Trial Management Group (TMG) will oversee the daily execution of the trial, while the Trial Steering Committee (TSC) will offer supervision. The TMG will include the principal investigator, research assistants (RAs), the Intervention Coordinator (IC), yoga instructors, and a representative of *Sahayoginis*.

There will be six members in the TSC and one of them will be a woman who has recently delivered a baby. She will represent patient perspectives and ensure that the safety of the participants is prioritized throughout the implementation of the trial. Two independent researchers will co-chair TSC. Details about the membership, roles and responsibilities, and TSC meetings are provided in the supplementary file ( *Extended Data* ^36^). Representatives from the TMG will participate in the TSC meetings.

***Composition of the data monitoring committee, its role and reporting structure***

Due to the nature of the trial and the low risk for participants, the TSC will also take on the role of the data monitoring committee. The Clinical Research Coordinator will submit a report every two weeks summarizing SAEs in both the arms without removing the blinding. During the TSC meetings, data related to trial progress and participants' safety will be reviewed and discussed.

***Adverse event reporting and harms***

The IC and yoga instructors will be trained to identify Serious Adverse Events (SAEs) and notify them to the CRC. CRC will maintain a log of all the following SAEs in both arms.

1) Death due to any cause
2) Hospitalization due to any cause
3) Suicide attempt
4) Spontaneous abortion or stillbirth

RAs will independently document SAEs at the three-month and post-delivery follow-up assessment. PI will submit an initial report to the Institutional Ethics Committee about the SAE (related or unrelated) within 24 hours of becoming aware. This will be followed up by a detailed analysis report submitted to the Institutional Ethics Committee within 14 days of the knowledge of the occurrence of the SAE. A standard operating procedure will include all the details about the detection, appropriate response, and reporting of SAEs. If any SAE requires an immediate response, an independent medical specialist will do so within two working days. A summary of all serious adverse events will be sent to the Institutional Ethics Committee, the funder, and the TSC regularly as part of the progress report. If there is a statistically significant difference in prevalence of SAEs in the two arms of the trial, the TSC will take a decision to unblind the data. All necessary and appropriate medical assistance will be provided to participants in the PIMS for injuries related to participation in the trial (intervention arm). There will be no financial burden on the participants for trial-related injuries and adverse events.

***Frequency and plans for auditing trial conduct***

The sponsor and the Institutional Ethics Committee will monitor the aspects of the study on an ongoing basis. PI will send a monthly progress report to the Institutional Ethics Committee.

***Plans for communicating important protocol amendments to relevant parties (e.g., trial participants, ethical committees)***

During the progress of the trial, we may have to undertake certain modifications in the protocol that may affect the scientific value of the study, participant safety, or how the study is conducted. PI will first submit these modifications to the Institutional Ethics Committee as amendments to the protocol and then seek approval from the TSC and the sponsor. We will communicate all the changes made to the protocol to the trial participants through written communication. The final report submitted to the funder will enlist all the amendments to the protocol.

### Sponsor/funder information

This work is funded by a grant from the DBT-Wellcome Trust India Alliance to the first author.

The funder did not contribute to the trial design and will not be involved in any aspect related to the implementation of the trial, such as data collection, management, analysis, or data interpretation. Additionally, the funder will not participate in writing the report or decisions regarding its submission for publication. Pravara Institute of Medical Sciences is the primary sponsor for the trial.

### Dissemination plans

Our research will primarily communicate key findings through publications in open-access peer-reviewed journals. Upon acceptance for publication, the final peer-reviewed journal manuscripts resulting from this research will be archived in PubMed Central (or an equivalent digital repository). PI and other study team members will present research data at national and international scientific conferences, including oral presentations, posters, and published abstracts, prior to formal publication. Comprehensive study reports containing result interpretations and recommendations will be shared with all major stakeholders. Findings will also be disseminated to participating communities through leaflets, flyers, local media, and interactive community meetings.

## Discussion

In this protocol paper, we provide the rationale, detailed description (using the TiDieR guidelines), and evaluation plan for assessing the feasibility, accessibility, and preliminary efficacy of Yoga-Sanskar, a yoga-based intervention for antenatal depression. Almost all studies evaluating the effect of yoga on depression during pregnancy have taken place either in high-income settings such as the United States and the United Kingdom or in urban settings in India ^7^. We need to understand more about women's uptake of yoga-based intervention in rural India. Previous trials have yet to undertake an economic evaluation of yoga-based intervention, and there is no data about the costs involved in the delivery/practice of the intervention. This pilot randomized controlled trial tries to address these critical research gaps.

We will recruit participants from the hospital setting (antenatal clinic of PIMS and antenatal clinics run by private obstetricians) and community settings. The latter's advantage is that women get more time to interact with the study team. This facilitates their decision to participate in the study compared to the hospital setting where they are more concerned about the delays in antenatal check-up due to interaction with the study team members. On the other hand, it is easier to approach a large number of pregnant women on a single day in hospital setting. We will screen all pregnant women for the current episode of depression, generalized anxiety disorder, and perceived stress. Thus, even if a pregnant woman is enrolled in the comparison arm of the study or is not eligible to participate in the study, she will at least get an assessment of her mental health status and a single health education session. Information related to sleep hygiene, diet, and physical activity will be delivered in this single health education session of about ten minutes using an educational leaflet. In a rural setting, the most critical challenge for a pregnant woman is to travel to any location far from her house to participate in any behavioral intervention. She also finds it difficult to take time out of her daily routine to practice these interventions. These were the key findings from our previous trial in the same geographical setting ^37^ and our formative research. Yoga-Sanskar intervention is designed in a way that the time commitment for enrolled women to attend supervised group yoga sessions is limited to three sessions, about five hours of their time outside the home. After completing these three sessions that constitute the LEARN phase, we will provide them with a journal and access to YouTube recordings to enable regular home practice of yoga. Yoga-instructors and *Sahayoginis* will regularly contact and motivate the participants to continue home practice. We are hopeful that these strategies will improve the adherence to the intervention and will have a positive effect on the outcomes. At the end of the LEARN phase, we will assess the competency of the participants to practice yoga independently and safely. This is a unique feature of our study as previous studies evaluating yoga-based intervention in pregnant women have not included competency assessment ^7^.

During the conduct of the trial, we will be cautious to ensure the safety of the participants. As noted earlier, who will recruit only those women who do not have history of spontaneous abortion, each supervised yoga session will begin with completion of the safety checklist, and the project team will keep a track of injuries documented by the participants every week.

A community representative and a yoga instructor have reviewed this manuscript and have agreed to be one of the co-authors. The protocol was also presented to all *Sahayoginis*, and their feedback has been incorporated.

Overall, this study will address an important research gap in the field of perinatal mental health and will set the stage for undertaking the explanatory trial.

## Ethics and consent

The Institutional Ethics Committee of the Pravara Institute of Medical Sciences (Approval Number: PIMS/DR/RMC/477) approved this study. Eligible participants who express willingness to participate in the study will provide informed written consent by completing a hard copy of the consent form.

## Data Availability

No data are associated with this article. Figshare: Participant Information Sheet. https://doi.org/10.6084/m9.figshare.25866205 ^38^. Figshare: Informed Consent forms. https://doi.org/10.6084/m9.figshare.25867051 ^10^. Figshare: Assessment Tools. https://doi.org/10.6084/m9.figshare.25866424 ^17^. Figshare: Trial Steering Committee_Terms of Reference. https://doi.org/10.6084/m9.figshare.25866523 ^36^. Data are available under the terms of the Creative Commons Attribution 4.0 International license (CC-BY 4.0). Figshare: SPIRIT checklist for ‘Yoga-based lifestyle intervention for Antenatal Depression (YOGA-D): study protocol for a pilot randomized controlled trial’. https://doi.org/10.6084/m9.figshare.25866463 ^9^ Data are available under the terms of the Creative Commons Attribution 4.0 International license (CC-BY 4.0).
