# Supplementary material for: Yoga-based lifestyle intervention for antenatal depression (YOGA-D): study protocol for a pilot randomized controlled trial
Source: Wellcome Open Res. 2024 Nov 6;9:326. Originally published 2024 Jun 21. [Version 2] doi: 10.12688/wellcomeopenres.22493.2 (PMC11574329; doi:10.12688/wellcomeopenres.22493.2)
Supplement: Peer review history

# Reviewer report: Reviewer response for version 2

**DOI:** 10.21956/wellcomeopenres.25719.r110339

**Published:** 2024-11-19

**Authors:** James Newham

**Affiliations:** 1 Northumbria University, Newcastle upon Tyne, England, UK

I am happy with the amendments.

Is the study design appropriate for the research question?

Yes

Is the rationale for, and objectives of, the study clearly described?

Partly

Are sufficient details of the methods provided to allow replication by others?

Yes

Are the datasets clearly presented in a useable and accessible format?

Yes

Reviewer Expertise:

I ran the first randomized controlled trial of antenatal yoga for mental health difficulties. At the moment the lack of clarity of the mental health status of the participants prevents me from approving but feel this can be addressed

I confirm that I have read this submission and believe that I have an appropriate level of expertise to confirm that it is of an acceptable scientific standard.

# Reviewer report: Reviewer response for version 2

**DOI:** 10.21956/wellcomeopenres.25719.r111772

**Published:** 2024-11-18

**Authors:** Carolina Estevao

**Affiliations:** 1 Kings College London, London, UK

The manuscript presents a valuable study addressing a critical gap in maternal health, particularly in underserved populations. However, several areas require further clarification and improvement:

 A few pointers:

1. The limitation to singleton pregnancies needs justification.
2. The exclusion of high-risk women, such as those with a history of self-harm or suicidal ideation, raises concerns. This group may benefit significantly from the intervention, and their exclusion should be justified.
3. The study does not consider using the Edinburgh Postnatal Depression Scale (EPDS), a validated tool for prenatal depression screening. Incorporating the EPDS could enhance the study's robustness and comparability with existing literature.
4. The assumption that prenatal yoga can address a broad range of minor ailments needs re-evaluation. It is important to discuss the limitations and specify which ailments are targeted based on existing evidence.
5. For participants who are illiterate, alternative methods for delivering intervention materials (QR code/youtube videos) should be considered.
6. The strategy for monitoring participants' mental health throughout the study is unclear. The manuscript should include a plan for regular mental health assessments and specify how participants will be supported if their mental health deteriorates.

In general, the study design is well designed, particularly the behavioral science approach. The intention to seek renewed consent at different stages of the study shows a strong ethical commitment.

 The focus on an underserved population addresses an important public health need, making this research highly relevant.

Is the study design appropriate for the research question?

Yes

Is the rationale for, and objectives of, the study clearly described?

Yes

Are sufficient details of the methods provided to allow replication by others?

Yes

Are the datasets clearly presented in a useable and accessible format?

Not applicable

Reviewer Expertise:

Yoga for maternal mental health

I confirm that I have read this submission and believe that I have an appropriate level of expertise to confirm that it is of an acceptable scientific standard.

# Reviewer report: Reviewer response for version 1

**DOI:** 10.21956/wellcomeopenres.24784.r94691

**Published:** 2024-10-02

**Authors:** Chuntana Reangsing

**Affiliations:** 1 Mae Fah Luang University, Mueang Chiang Rai, Chiang Rai, Thailand

Dear Editor,

 Thank you so much for giving me opportunities to review this paper.

 I have some comments. See below.

- Prenatal yoga has conceptual framework as yoga-Sanskar or not? What is the difference between these types of yoga. To better understand for new audiences if the researcher provide the concept of Yoga-Sanskar in introduction

Overall, the paper was well-written and clearly reported in terms of concepts and methodologies and well organized. Thus, I would recommend this paper to be Indexed.

Is the study design appropriate for the research question?

Yes

Is the rationale for, and objectives of, the study clearly described?

Yes

Are sufficient details of the methods provided to allow replication by others?

Yes

Are the datasets clearly presented in a useable and accessible format?

Yes

Reviewer Expertise:

i am expert in alternative medicine and statistical test.

I confirm that I have read this submission and believe that I have an appropriate level of expertise to confirm that it is of an acceptable scientific standard.

# Response

**Published:** 2024-10-25

**Authors:** Rahul Shidhaye

**Affiliations:** Pravara Institute of Medical Sciences, India

**24 October 2024**

 Dear Dr. Reangsing,

**Re: Manuscript title: Yoga-based lifestyle intervention for antenatal depression (YOGA-D): study protocol for a pilot randomized controlled trial**

 Many thanks for taking out time to review our submission and providing very valuable comments and suggestions. We have responded to each of your comments in italics below and believe we have been able to address all the comments. We have made corresponding changes to the manuscript and the revised manuscript is uploaded.

**Comment # 1:** Prenatal yoga has conceptual framework as yoga-Sanskar or not? What is the difference between these types of yoga. To better understand for new audiences if the researcher provide the concept of Yoga-Sanskar in introduction

**Response:** *Thank you so much for your suggestion. We have used the term ‘prenatal yoga’ in a generic way. This term (as used in our manuscript) does not refer to any specific yoga program. We are not aware of any trademark or branded ‘prenatal yoga’ intervention. As per our understanding, yoga-based intervention practiced during the antenatal period is ‘prenatal yoga’. In the manuscript we have explicitly mentioned that ‘Yoga-Sanskar’ is a type of gentle, pre-natal yoga-based intervention.* *We agree with your suggestion about describing concept of Yoga-Sanskar introduction.* *We have provided the detailed description, including the rationale for the same in the section titled, ‘ **Intervention arm: Yoga-Sanskar intervention for antenatal depression’. **We have followed Template for Intervention Description and Replication (TIDieR) guidelines to describe the intervention. TIDieR document is uploaded to Figshare ( https://doi.org/10.6084/m9.figshare.25866484 ).*

**Comment # 2:** Overall, the paper was well-written and clearly reported in terms of concepts and methodologies and well organized. Thus, I would recommend this paper to be Indexed. **Response:** *Thank you so much for your positive feedback. We really appreciate it.  *      

# Reviewer report: Reviewer response for version 1

**DOI:** 10.21956/wellcomeopenres.24784.r89010

**Published:** 2024-08-06

**Authors:** James Newham

**Affiliations:** 1 Northumbria University, Newcastle upon Tyne, England, UK

The study protocol is very thorough and the list of measures is comprehensive. I would be very interested in the outcome of this trial and feel it is an important area. My comments now are primarily regarding clarity/points for improvement; and do not undermine the design of the study. However the clarity on the participant group is essential for approval.

 I mention ‘partly’ for Objectives as I’m unsure of the cohort of participants you are trying to focus on as while it states depression, the inclusion/exclusion criteria is confusing. It states that participants must score over 10 on the PHQ which is considered ‘moderate depression’ (on this scale people can score ‘None, ‘Mild depression’, ‘Moderately severe’ and ‘Severe’). However the exclusion criteria states that ‘receiving treatment for depression or any other mental health condition’ is an exclusion criteria. This strikes me that you are potentially identifying and recruiting women at risk who should be receiving additional treatment. You state in the Discussion that participants ‘will at least get an assessment of her mental health status and appropriate psychoeducation’ but no other mention to ‘psychoeducation’ is made. I feel you would be better trying to recruit those scoring with ‘mild depression’ as there is less clinical rationale to refer them for additional treatment or alternatively allow women who are receiving treatment. At the moment the idea of identifying/recruiting a high risk group without referral is problematic.

**Major considerations:**

- There are a lot of components in the intervention that go beyond yoga (e.g. info on physical activity, sleep and diet) and not sure how you are going to isolate them from the effective components of the interventions. Maybe something to enquire about in the interviews? It would help to clearly outline what both groups get.
- There is a wide gestational age (12-26 weeks) for inclusion and if assessments are done 3 months post-randomisation then some will already be at term (37 weeks) – so feel you will miss some and with a small group I think this could be problematic. In addition, this does create a very heterogenous group and so potentially you are going to have people scoring very differently simply by being at such divergent gestational ages. For example, the early starters will be assessed at the baseline gestational age of the late starters.  
- I was confused about the postpartum period delivery of an additional yoga course. This seems a study in itself and may impact when the post-delivery assessment is performed. It’s a nice idea but I think the EUC is enough and the postpartum yoga course seems to an added burden that doesn’t help isolate impact of the comparison you already have.

**Minor considerations:**

- “We will approach obstetricians practicing in the above towns and if they agree then participants will be recruited from these private antenatal clinics.” I think it is important to collect this data as is important feasibility data.
- Likewise record the number excluded due to ‘Inability to attend in-person yoga sessions’ as important feasibility metric
- How will you monitor practice at home – this will cause a dosage effect making differences within the intervention group
- Anganwadi – this term is not explained

Is the study design appropriate for the research question?

Yes

Is the rationale for, and objectives of, the study clearly described?

Partly

Are sufficient details of the methods provided to allow replication by others?

Yes

Are the datasets clearly presented in a useable and accessible format?

Yes

Reviewer Expertise:

I ran the first randomised controlled trial of antenatal yoga for mental health difficulties. At the moment the lack of clarity of the mental health status of the participants prevents me from approving but feel this can be addressed

I confirm that I have read this submission and believe that I have an appropriate level of expertise to confirm that it is of an acceptable scientific standard, however I have significant reservations, as outlined above.

# Response

**Published:** 2024-10-25

**Authors:** Rahul Shidhaye

**Affiliations:** Pravara Institute of Medical Sciences, India

**24 October 2024** Dear Dr. Newham,

**Re: Manuscript title: Yoga-based lifestyle intervention for antenatal depression (YOGA-D): study protocol for a pilot randomized controlled trial**

 Many thanks for taking out time to review our submission and providing very valuable comments and suggestions. We have responded to each of your comments in italics below and believe we have been able to address all the comments. We have made corresponding changes to the manuscript and the revised manuscript is uploaded.

**Comment # 1:  T**he study protocol is very thorough, and the list of measures is comprehensive. I would be very interested in the outcome of this trial and feel it is an important area. My comments now are primarily regarding clarity/points for improvement; and do not undermine the design of the study. However, the clarity on the participant group is essential for approval.

******Response:** *Thank you so much for your positive feedback. We really appreciate it.*

**Comment # 2:** I mention ‘partly’ for Objectives as I’m unsure of the cohort of participants you are trying to focus on as while it states depression, the inclusion/exclusion criteria is confusing. It states that participants must score over 10 on the PHQ which is considered ‘moderate depression’ (on this scale people can score ‘None, ‘Mild depression’, ‘Moderately severe’ and ‘Severe’). However the exclusion criteria states that ‘receiving treatment for depression or any other mental health condition’ is an exclusion criteria. This strikes me that you are potentially identifying and recruiting women at risk who should be receiving additional treatment. You state in the Discussion that participants ‘will at least get an assessment of her mental health status and appropriate psychoeducation’ but no other mention to ‘psychoeducation’ is made. I feel you would be better trying to recruit those scoring with ‘mild depression’ as there is less clinical rationale to refer them for additional treatment or alternatively allow women who are receiving treatment. At the moment the idea of identifying/recruiting a high risk group without referral is problematic.

**Response:**

- *We completely agree with you that, ‘identifying and recruiting pregnant women who are at high risk’, i.e., who are having severe depression and not providing them referral/specialist care is problematic.  *
- *Hence, we are excluding pregnant women who express death wish/ suicidal thoughts almost every day (score of 3 on PHQ9 item 9) and referring them to psychiatrist for specialist care. *
- *While drafting the protocol, we also felt that we should exclude pregnant women with severe depression (PHQ-9 score>19). However, we didn’t include that as a separate exclusion criterion as we felt that PHQ-9 item 9 (related to death wish and suicidal thoughts) score of greater than 2 would take care of this. *
- *Till date 34 RCTs have demonstrated utility of yoga in depression (https://www.ncbi.nlm.nih.gov/pmc/articles/PMC10077871/). Trials have also safely and effectively used yoga as a monotherapy in mild to moderate cases of depression ( https://pubmed.ncbi.nlm.nih.gov/28301561/ ; https://pubmed.ncbi.nlm.nih.gov/37883245/ ; https://pubmed.ncbi.nlm.nih.gov/29614706/)*
- *Our overall aim is to assess the effectiveness of a stand-alone yoga-based intervention for moderate and moderately severe antenatal depression. We are not assessing the benefits/effects of a yoga-based intervention as an additional treatment. So, we decided not to include pregnant women who are already receiving treatment for depression. *
- *Pregnant women are not screened for antenatal depression in the study area as part of the routine antenatal care visits. As a result, till now we have not come across any pregnant woman who is screen positive for depression (PHQ9>9) and is receiving treatment for depression. In the first six weeks of the pilot randomized controlled trial enrolment phase, we haven’t excluded any pregnant women based on the criterion of ‘receiving treatment for depression’. *
- *As mentioned earlier, we agree with your suggestion and based on the same we plan to exclude pregnant women with severe depression (PHQ9>19) in the larger follow-up pragmatic randomized controlled trial. In the protocol for the pragmatic randomized controlled trial, we will specifically mention that we wish to assess the effectiveness of a stand-alone yoga-based intervention on moderate and moderately severe antenatal depression. *
- *We have made following change in a sentence in the discussion section based on your comment related to psychoeducation. The included text is as follows, *

*‘Thus, even if a pregnant woman is enrolled in the comparison arm of the study or is not eligible to participate in the study, she will at least get an assessment of her mental health status and a single health education session.’ Information related to sleep hygiene, diet, and physical activity will be delivered in this single health education session of about ten minutes using an educational leaflet.* *We have replaced the term ‘psychoeducation’ with ‘single health education session’. The description of this session is in the response to comment # 3.*

**Comment # 3: ** There are a lot of components in the intervention that go beyond yoga (e.g. info on physical activity, sleep and diet) and not sure how you are going to isolate them from the effective components of the interventions. Maybe something to enquire about in the interviews? It would help to clearly outline what both groups get.

**Response:**

- *During formative research, we found that many pregnant women had issues related to sleep and wanted to know what they should or should not eat during pregnancy. *
- *Hence, we thought that it will be a good idea to provide very brief information about sleep and diet during pregnancy to trial participants in both the intervention arm and the comparison arm. *
- *Additionally, we decided to include brief information about physical activity during pregnancy for the participants in the comparison arm. This will ensure that comparison arm participants undertake some physical activity and that the difference between the two arms is not only because of presence or lack of physical activity in one arm compared to the other. *
- *Inclusion of this information for participants in the comparison arm will ensure that there is some ‘enhancement’ to the usual/routine care. *
- *Information related to sleep hygiene, diet, and physical activity is delivered in a single health education session of about ten minutes using an educational leaflet. This is now included in the description of the comparison arm. *
- *There are 12 simple instructions for getting good sleep (e.g., avoid sleeping for long hours during daytime), 13 diet related instructions (e.g., have small and frequent meals, include green leafy vegetables, citrus fruits in diet), and nine instructions related to physical activity during pregnancy (e.g., every pregnancy is different even for the same woman, so undertake only those physical activities that you are comfortable with). *
- *We have now uploaded the educational leaflet on figshare (https://doi.org/10.6084/m9.figshare.27300849).*
- *In nutshell, we would like to clarify that the information related to sleep hygiene, diet, and physical activity will be given to participants in both the arms. The information is brief and can’t be really termed as intervention component. *
- *We agree with your suggestion to include this aspect in our in-depth interviews that are planned to be conducted at the end of three months post-randomization and explore the role of this information on their sleep, diet, and physical activity. *

**Comment # 4: ** There is a wide gestational age (12-26 weeks) for inclusion and if assessments are done 3 months post-randomisation, then some will already be at term (37 weeks) – so feel you will miss some and with a small group I think this could be problematic. In addition, this does create a very heterogenous group and so potentially you are going to have people scoring very differently simply by being at such divergent gestational ages. For example, the early starters will be assessed at the baseline gestational age of the late starters.  

**Response:**

- *We agree with you that this is a major concern. *
- *While designing the study, our plan was to include women throughout the duration of pregnancy and study the effect of a yoga-based intervention on their depression. *
- *In our study setting, women generally tend to register with the health system by tenth to twelfth week of pregnancy. Women who wish to terminate pregnancy, generally do so by twelfth week. *
- *After completing six months of pregnancy (26 weeks), women generally go to their mother’s home for delivery. *
- *The period between 12 and 26 weeks is the time when they are in regular contact with the local health system. *
- *If we restrict this to 20 to 24 weeks (or any other shorter duration), many women will get excluded at the time of screening and it then becomes logistically difficult to contact them again and assess their eligibility for the study. *
- *Once the pilot randomized controlled trial is over, we plan to initiate a pragmatic randomized controlled trial with a total sample size of 336 pregnant women with depression (169 pregnant women with depression in each arm). *
- *This was essentially our rationale for the mentioned gestational duration.  *
- *We agree with you that divergent gestational age will create a heterogenous group. We will address this by including gestational age as one of the independent variables in the regression models and by doing a sub-group analysis of women with gestation age of 12 to 18 weeks and 19 to 26 weeks. Limitations arising due to small sample sizes will be included in the outcomes paper. Thus, gestational age at the time of recruitment will be documented and will serve as a covariate in data analysis. *

**Comment # 5:** I was confused about the postpartum period delivery of an additional yoga course. This seems a study in itself and may impact when the post-delivery assessment is performed. It’s a nice idea but I think the EUC is enough, and the postpartum yoga course seems to an added burden that doesn’t help isolate impact of the comparison you already have.

**Response:**

- *During our previous trial with pregnant women in the same study setting *(Shidhaye et al., 2023) *and the formative research work, we observed that pregnant women are generally keen to practice yoga during pregnancy.*
- *When pregnant women agree to participate in the study, they are generally under the impression that they will be practicing yoga during pregnancy. *
- *It is difficult for them to understand the concept of ‘randomization’ and in our experience many women want to be in the intervention arm. *
- *Most of the participants feel bad when they get allocated to the comparison arm. *
- *Hence, we decided to teach yoga to participants in the comparison arm about six weeks post-delivery (as wait-list controls). *
- *We plan to conduct post-delivery assessments four weeks after delivery. These assessments will not be affected by yoga taught to the comparison arm participants later.  *
- *We completely agree with you that yoga course in the postpartum period is an additional burden and that the EUC is enough. *
- *However, as a contribution to the community and to conform with the ethical requirements of research, our yoga instructors are happy to teach them yoga after the trial is over. *

**Comment # 6:** “We will approach obstetricians practicing in the above towns and if they agree then participants will be recruited from these private antenatal clinics.” I think it is important to collect this data as is important feasibility data.  

**Response:** *Thank you. We will certainly report this in the findings of the pilot randomized controlled trial. *  

**Comment # 7:   ** Likewise record the number excluded due to ‘Inability to attend in-person yoga sessions’ as important feasibility metric

**Response:** *Thank you. We will certainly report this in the findings of the pilot randomized controlled trial. *  

**Comment # 8: **   How will you monitor practice at home – this will cause a dosage effect making differences within the intervention group

**Response:** *As we have described in the description of the Yoga-Sanskar intervention, we will provide Yoga Journal (Yoga Dainandini) to participants in the active intervention arm. Yoga Journal will contain a detailed day-to-day plan for home practice and participants are expected to enter the amount of time they spend each day for the yoga practice. Participants can also mark which yoga activities they practiced each day.*

**Comment # 9:** Anganwadi – this term is not explained

**Response:** *Thank you for this suggestion. We have added the following information about the Anganwadi centres and Anganwadi workers in the section on eligibility criteria in the revised version of the manuscript,* *‘* *Anganwadi* *is a community-based center providing healthcare check-up and referral health services, supplementary nutrition, immunization, and non-formal pre-school education. Expectant and nursing mothers, children, and adolescent girls are the primary beneficiaries. These centers were established as part of the Integrated Child Development Service (ICDS) and are managed by female community health workers called Anganwadi workers. Each center caters to a population of approximately 1000 people in rural areas. Anganwadi workers have an updated list of all pregnant women in their respective areas.’*   Shidhaye, R., Bangal, V., Bhargav, H., Tilekar, S., Thanage, C., Gore, S., Doifode, A., Thete, U., Game, K., Hake, V., & Kunkulol, R. (2023). Feasibility, acceptability, and preliminary efficacy of yoga to improve maternal mental health and immune function during the COVID-19 crisis (Yoga-M2 trial): a pilot randomized controlled trial. *Frontiers in Human Neuroscience*, *17*. https://doi.org/10.3389/fnhum.2023.1115699      
